# Ganglioneurofibroma arising within the extralobar pulmonary sequestration

**DOI:** 10.1186/s13019-020-01295-9

**Published:** 2020-09-11

**Authors:** Yuanyuan Liu, Wenbin Wu, Longbo Gong, Miao Zhang

**Affiliations:** 1 Department of Respiratory and Critical Care Medicine, Xuzhou Central Hospital, Xuzhou, China; 2 Department of Thoracic Surgery, Xuzhou Central Hospital, 199 Jiefang South Road, Xuzhou, 221009 China

**Keywords:** Pulmonary sequestration (PS), Three-dimensional computed tomography angiography (3D-CTA), Uniportal, Single port, Video-assisted thoracoscopic surgery (VATS)

## Abstract

**Background:**

Neurogenic tumor arising within the pulmonary sequestration (PS) is rare.

**Case presentation:**

A 42-year-old asymptomatic female was referred to our hospital for work-up of extralobar PS. The independent feeding artery from the thoracic aorta was confirmed by three-dimensional computed tomography angiography (3D-CTA). Uniportal thoracoscopic resection of the sequestrated lung with mediastinal lymph node sampling was performed successfully. Ganglioneurofibroma within the PS was diagnosed as the specimen revealed positive expression of SRY-related HMG-box 10 protein, neuron-specific enolase, S-100, chromogranin A and synuclein. Tumor recurrence was not recorded 1 year after the surgery.

**Conclusion:**

Preoperative 3D-CTA is useful to identify the aberrant vessels of PS. An elaborate diagnostic work-up after a timely resection is necessary for subsequent management and follow-up plan.

## Background

Ganglioneuroma is a rare, benign and well-differentiated neurogenic tumor, which is mostly localized in the posterior mediastinum [1]. It usually grows very slow and displaces the surrounding anatomical structures without infiltration [2]. To date, no specific serum biomarkers have been established for the diagnosis of neurogenic tumors. Pulmonary sequestration (PS) is mainly defined as a non-functioning lung tissue that has an unusual feeding artery mostly arising from the aorta, without certain pathological diagnostic criteria [3]. The etiology of PS is unknown. To our knowledge, neurogenic tumor originated in the sequestered lung is rare. Herein we presented a case of ganglioneurofibroma arising within an extralobar PS, followed by a brief literature review.

## Case presentation

**Fig. 1:**
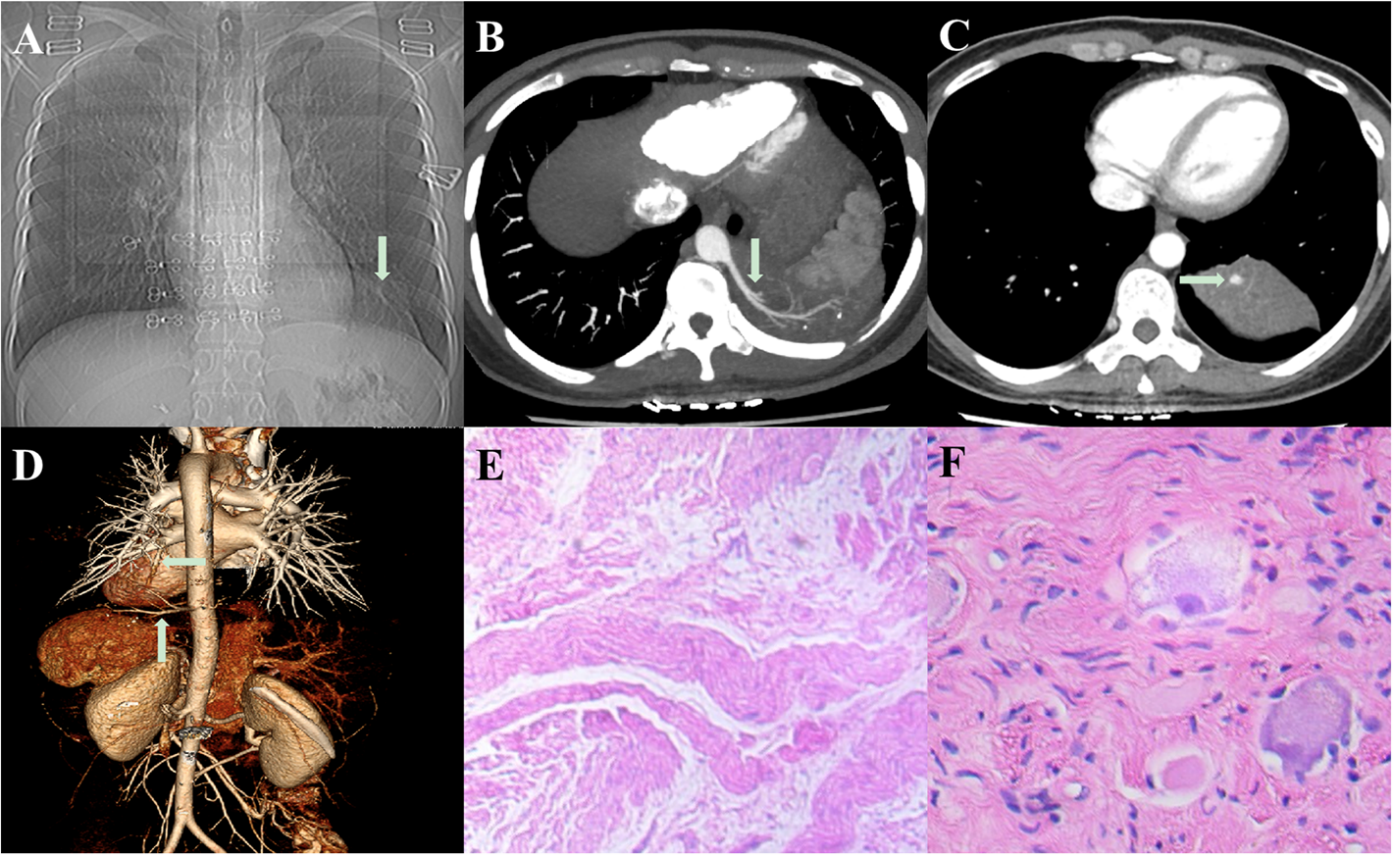
**a** The chest X-ray revealed a mass in the left thorax; **b** The CT showed that the mass was located in the posterior mediastinum with a feeding artery (indicated by the arrow) from the aorta; **c** The effluent vein within the lesion was noticeable (indicated by the arrow). **d** The 3D-CTA revealed the feeding artery (indicated by the vertical arrow) and the effluent vein (indicated by the horizontal arrow). **e** Frozen-section staining showed the diagnosis of ganglioneurofibroma (hematoxylin-eosin staining, × 40). **f** Magnified pathological image confirmed the ganglioid cells and Schwann cells (hematoxylin-eosin staining, × 400)

This report was approved by the Institutional Review Board of Xuzhou Central Hospital, and written informed consent was obtained from the patient. The clinical data was presented anonymously for privacy concern. A 42-year-old asymptomatic female nonsmoker was admitted in June 2019 because the chest x-ray indicated a shadow in the left thorax (Fig. 1a). The blood tests showed that the tumor biomarkers such as carcinoembryonic antigen, alpha-fetoprotein, neuron-specific enolase, and cytokeratin-19 fragment were all in normal range. Further contrast-enhanced computed tomography (CT) revealed a homogenous mass located in the posterior mediastinum with a feeding artery from the thoracic aorta and an effluent vein into the left inferior pulmonary vein (Fig. 1b and c).

Based on these findings, an extralobular PS was empirically diagnosed. Uniportal thoracoscopic resection of the sequestrated lung was scheduled and performed after a multidisciplinary evaluation. A thorough preoperative work-up including abdomen CT, and whole-body bone emission CT was conducted, which excluded other suspicious lesions. Meanwhile, three-dimensional CT angiography (3D-CTA) was utilized [4], which clearly demonstrated the anomalous vessels including the feeding artery and the effluent vein of the PS (Fig. 1d).

Fast-track surgery protocol was utilized for this case. The patient underwent surgery under general anesthesia with one-lung ventilation in a right lateral decubitus position. A 10 mm, 30 degree thoracoscope was inserted into the left pleural cavity through a 3.5 cm single incision in the 6th intercostal space along the anterior axillary line. The aberrant artery about 7 mm in diameter and the effluent vein about 5 mm in diameter within the sequestrated lung were identified and transected using a 45-mm stapling device, respectively. The PS was removed completely. The frozen-section staining of the specimen revealed the diagnosis of ganglioneurofibroma (Fig. 1e). The operation time was 70 min, while the estimated blood loss was about 20 mL. R0 resection was achieved while the sampled mediastinal lymph nodes were tumor-negative. Ultrasound-guided serratus anterior plane block using a bolus of liposomal bupivacaine was used for analgesia. In addition, postoperative chest tube drainage was avoided.

Ganglioneurofibroma arising within the extralobar PS (Fig. 1f) was confirmed by pathological staining as well as immunohistochemistry tests of the specimen which indicated positive expression of SRY-related HMG-box 10 protein, neuron-specific enolase, S-100, chromogranin A and synuclein. The patient displayed an uneventful course and was discharged from the hospital on postoperative day 3. During the 1 year follow up, the patient reported satisfactory quality of life while residual pleural effusion or tumor recurrence was not identified.

## Discussion and conclusions

The coexistence of lung malignancy and PS is extremely rare. The present case revealed synchronous primary ganglioneurofibroma arising within the extralobar PS, and the outcome was satisfactory after a timely radical resection of the sequestrated lung. Although chest radiographs can indicate the specific findings suspicious of PS in most cases, 3D-CTA is the preferred imaging modality for identifying the aberrant feeding arteries and effluent veins of the sequestrated lung, which is of vital importance to diminish iatrogenic massive hemorrhage in the operation.

Thoracic neurogenic neoplasms may originate from any nervous structure in any mediastinal compartment or in the chest wall due to the complex anatomy of the nervous system [5]. Schwannoma and neurofibroma represent the most common mediastinal neurogenic tumors that rarely degenerate into malignant tumors; whereas the sympathetic ganglia tumors include benign ganglioneuroma, malignant ganglioneuroblastoma, and neuroblastoma [6]. The treatment options for neurogenic tumors vary depending on the presentation, but most often the surgical resection is recommended although the patients with malignant neurogenic tumors still have poor long-term survival prospects [7]. Nearly one-half of adult PS patients are asymptomatic [8]; however, a timely resection should always be considered as the optimal treatment. In theory, preoperative embolization of the aberrant blood supply in the PS may mitigate the risk of intraoperative bleeding; however, our present case confirmed the safety of uniportal thoracoscopic surgery without vascular intervention. Similarly, biopsy and staging are not appropriate when a radical resection could be achieved. The objective of a timely resection is to facilitate the differential pathological diagnosis [9].

We searched PubMed, Web of Science, Scopus, Embase, Europe PMC, Cochrane Library and Google Scholar for similar reports up to January 2020. Key words and MeSH terms in title or abstract including “pulmonary sequestration” and “concurrent” or “synchronous” and “tumor” were used. No restriction was made regarding the publication language. Finally a total of 12 case reports were summarized and listed in Table 1. These cases demonstrate the probability of PS in the differential diagnosis for asymptomatic mediastinal masses involving the adjacent lung or mediastinum. The tumor findings were reported in both intralobar and extralobar PS; whereas most of the patients were admitted due to non-specific manifestations ranging from cough, recurrent hemoptysis to pneumonia. Surgical resection is recommended as the first treatment option for PS to avoid repeated infection, recurrent hemorrhage and potential primary or secondary malignancy. Furthermore, a timely resection might provide a satisfactory prognosis.

**Table 1:** Previous reports of tumor arising within the pulmonary sequestration

Author, year	Age	Gender	Smoking history	Type of sequestration	Manifestation on admission	Tumor type	Treatment	Prognosis
Hertzog, 1963 [10]	NR	NR	NR	NR	NR	Epidermoid cancer	NR	NR
Bell-Thomson, 1979 [11]	69	Male	NR	Intralobar	NR	Squamous cell carcinoma	NR	NR
Peroś, 1980 [12]	NR	NR	NR	NR	NR	Cancer	NR	NR
Juettner, 1985 [13]	45	Male	NR	Intralobar	NR	Bronchial carcinoid	Lobectomy	7 years, alive
Gatzinsky, 1988 [14]	50	Female	Smoker	Intralobar	Improductive cough	Carcinoma	Lobectomy	NR
Morita, 1994 [15]	59	Male	NR	Intralobar	Fever	Squamous cell carcinoma	Segmentectomy	10 months, alive
Hekelaar, 2000 [16]	31	Female	Nonsmoker	Intralobar	Digital clubbing and coughing	Primary lymphoepithelioma-like carcinoma	Pneumectomy after recurrence within 2 years after the initial thoracotomy	4 years, alive
Ahmetoğlu, 2003 [17]	2	Girl	None	Extralobar	NR	Sclerosing haemangioma	NR	NR
Simoglou, 2015 [18]	67	Male	Smoker	Intralobar	Hemoptysis	Adenocarcinoma	Lobectomy	28 months, NR
Sato, 2015 [19]	67	Female	Nonsmoker	Extralobar	Asymptomatic	Ectopic ACTH-producing pulmonary carcinoid	Surgery	The serum ACTH was normal
Mengoli, 2016 [20]	34	Male	Nonsmoker	Extralobar	Recurrent hemoptysis	Malignant pigmented perivascular epithelioid cell neoplasm	Surgery	5 months, alive
Kayawake, 2020 [21]	50	Female	NR	Intralobar	Asymptomatic	Adenocarcinoma	Wedge resection	20 months, alive
The present case	42	Female	Nonsmoker	Extralobar	Asymptomatic	Ganglioneurofibroma	Resect the sequestered lung	12 months, alive

Abbreviations: *ACTH* adrenocorticotrophic hormone; *NR* not reported

In summary, we presented a case of ganglioneurofibroma which originated within the extralobar PS. Uniportal thoracoscopic surgery assisted with 3D-CTA is safe for extralobar PS; while a thorough pathological diagnosis of the sequestrated lung is necessary for subsequent management plan.

## Data Availability

The data of the present case is available from the corresponding author on reasonable request.

## Abbreviations

PS: Pulmonary sequestration
CT: Computed tomography
3D-CTA: Three-dimensional CT angiography
VATS: Video-assisted thoracoscopic surgery
